# Supplementary material for: Asynchronous magnetic resonance elastography: Shear wave speed reconstruction using noise correlation of incoherent waves
Source: Magn Reson Med. 2022 Oct 27;89(3):990–1001. doi: 10.1002/mrm.29502 (PMC9792433; doi:10.1002/mrm.29502)
Supplement: Supplementary file 1 — DATA S1 MATLAB code used to generate the numerical simulations of Figure 1 [file MRM-89-990-s002.zip › k-Wave/helpfiles/fwhm.html]

fwhm :: Functions (k-Wave)


# fwhm

Compute the full width at half maximum.

## Syntax

```
fwhm_val = fwhm(f, x)
fwhm_val = fwhm(f, x, plot_fwhm)
```

## Description

`fwhm` calculates the Full Width at Half Maximum (FWHM) of a positive 1D input function f(x) with spacing given by x.

For example, running the code

```
x = -pi/2:pi/20:pi/2;
f = cos(x); 
fwhm(f, x, true);
```

produces the output

## Inputs

|  |  |
| --- | --- |
| `f` | f(x) |
| `x` | x data or dx |

## Optional Inputs

|  |  |
| --- | --- |
| `plot_fwhm` | Boolean controlling whether a plot of the function and FWHM is produced |

## Outputs

|  |  |
| --- | --- |
| `fwhm_val` | FWHM of f(x) |
